# Translocation of mitochondrial DNA into the nuclear genome blurs phylogeographic and conservation genetic studies in seabirds

**DOI:** 10.1098/rsos.211888

**Published:** 2022-06-15

**Authors:** Torres Lucas, Bretagnolle Vincent, Pante Eric

**Affiliations:** ^1^ Centre d'Etudes Biologiques de Chizé, UMR 7372, CNRS - La Rochelle Universite, Villiers en Bois, France; ^2^ Littoral, Environnement et Sociétés, UMR 7266 CNRS - La Rochelle Université, La Rochelle, France

**Keywords:** pseudogenes, mitochondrial DNA, numts, mito-nuclear discordance, transposable elements, shearwater

## Abstract

Mitochondrial DNA (mtDNA) translocated into the nuclear genome (numt), when co-analysed with genuine mtDNA, could plague phylogeographic studies. To evaluate numt-related biases in population genetics parameters in birds, which are prone to accumulating numts, we targeted the mitochondrial *mt-cytb* gene. We looked at 13 populations of Audubon's shearwater (*Puffinus lherminieri*), including five mitochondrial lineages. *mt-cytb* homologue and paralogue (numt) sequences were determined by Sanger sequencing with and without prior exonuclease digestion of nuclear DNA. Numts formed monophyletic clades corresponding to three of the five mitochondrial lineages tested (the remaining two forming a paraphyletic group). Nineteen percent of numt alleles fell outside of their expected mitochondrial clade, a pattern consistent with multiple translocation events, incomplete lineage sorting (ILS), and/or introgression. When co-analysing *mt-cytb* paralogues and homologues, excluding individuals with ambiguities underestimates genetic diversity (4%) and differentiation (11%) among least-sampled populations. Removing ambiguous sites drops the proportion of inter-lineage genetic variance by 63%. While co-analysing numts with mitochondrial sequences can lead to severe bias and information loss in bird phylogeographic studies, the separate analysis of genuine mitochondrial loci and their nuclear paralogues can shed light on numt molecular evolution, as well as evolutionary processes such as ILS and introgression.

## 1. Introduction

Mitochondrial DNA (mtDNA) is the most popular marker for the study of molecular diversity in animals [1–3]. Reasons include the ease with which mtDNA is amplified, being 100–1000 times more abundant than genomic DNA [4,5], the levels of variation allowing us to compare its signal within and across animal taxa [6], and the fact that these genes are single copy with no introns (but see [7] and [8] for counterexamples), with short intergenic regions [9]. MtDNA has played a major role in the study of evolution with thousands of studies per year published since 1998 (see [10] for a review). However, the use of mtDNA as a marker of evolutionary history also has some drawbacks. The uniparental mode of inheritance of mitochondria, from mother to offspring, allows only the genetic history and structure of female lineages to be investigated [1]. In addition, mtDNA can recombine and is not universally maternally inherited (e.g. [11,12]). Recombination has been detected in several taxa (see [13] for a review) as well as copies of mitochondrial markers in the nuclear genomes [14], the so-called nuclear-mitochondrial pseudogenes or numts [15]. Heteroplasmy, i.e. the presence of several different mitogenomes within a single individual, may result from a biparental (or doubly uniparental, in the case of gonochoric bivalves) transmission of mitochondria (e.g. [11,12]). Finally, some mtDNA markers appear to be duplicated within the mitogenome of several species (e.g. in birds [16], salamanders [17] and lizards [2]). Whatever their causal origin, such multiple copies may affect sequence data interpretation, as they can be co-amplified by PCR. Alternatively, if only one of the copies is amplified, we may not know which one, and it may not necessarily be the same for all individuals. The presence of paralogues can therefore blur analyses of mtDNA genetic diversity, divergence and differentiation among populations. Numts, duplication and heteroplasmy have been found repeatedly in many taxa, including birds [16,18]. As bird blood is particularly poor in mitochondria [18], numts can easily be co-amplified along with mtDNA [18].

Petrels and albatrosses (order Procellariiformes) are seabirds for which mitochondrial DNA have been—and still is—extensively used to study phylogenetics, biogeography and systematics of the group, in spite of its complex evolutionary history. Abbott *et al.* [19] first described a duplicated region including *mt-cytb*, *mt-nd6*, the Control Region (CR) and multiple tRNAs within the mitogenome of five species of albatross. Other Procellariformes species were since found to have mitogenomes with duplications [19–23]. In this taxon, the duplicated genes can therefore not be PCR-amplified and Sanger-sequenced directly without risking amalgamating paralogues. In addition, the taxonomic distribution of this mitochondrial gene duplication event is not well known. Despite this issue, fewer than 30% (13/45) of subsequent papers took it into consideration (electronic supplementary material, table S1 summarizing studies published after 2005 and using mitochondrial markers to study the evolutionary biology and molecular systematics of Procellariiformes). Aside from the issue of intra-chromosomal duplication events, 14 studies mentioned the likely co-amplification of numts along with mtDNA and five mentioned heteroplasmy as a source of ambiguities (electronic supplementary material, table S1). Interestingly, intra-mitochondrial gene duplication is generally discussed for the CR, while numts are discussed when *mt-cytb* is considered, even though the CR can be translocated in the nucleus and *mt-cytb* can be included in the mitochondrial duplication [19]. Reported strategies to deal with the issues of duplication, numts and heteroplasmy are multiple. Authors dealing with CR duplication either removed mitochondrial CR data from analyses or used copy-specific PCR primers. Authors reporting ambiguities either designed taxon-specific primers [24], removed the sites presenting ambiguities [25] or removed the individual sequences that presented ambiguities [26]. These strategies may have major impacts on the estimation of genetic diversity, divergence and differentiation within and among populations. It has been shown in insects that *cox1* numts showed a high degree of divergence with mitochondrial sequences [27] artificially raising the diversity and the number of estimated species [28] and blurring the phylogenetic signal [29]. However, the impact of different treatments of multiple copies on statistics used in phylogeography and conservation genetics has never been quantified to our knowledge.

In this study, we use the petrel species complex *Puffinus lherminieri*, in which all three problems occur simultaneously [23,30], as a worst-case scenario to investigate the effects of amalgamating mitochondrial loci and their paralogues when attempting to estimate genetic diversity, divergence and differentiation within and among populations. Numts corresponding to several mitochondrial loci were recently detected in this group [30]. Moreover, this species is one of the many Procellariiformes that show a duplicated region in the mitochondrial genome [19–23]. Finally, full mitogenome sequencing suggested the possibility of heteroplasmy in this species [23]. Our first aim consisted of evaluating the impact of these multiple copies on population-level statistics routinely used in genetic analyses, while our second aim consisted of evaluating to which extent numts affect the evolutionary scenarios obtained from mitochondrial sequences. We chose to target cytochrome-b (*mt-cytb*) because this mitochondrial gene is informative at the phylogeographic scale in this group of shearwaters [30] and has been a marker of choice for many seabird species (e.g. electronic supplementary material, table S1). Also, *mt-cytb* was translocated to the nucleus, yielding numts [30], but is not duplicated in the mitogenome of *P. lherminieri* [23]; intra-individual polymorphisms can therefore be attributed to the co-amplification of numts rather than other types of mitochondrial paralogues. We thus generated three different datasets: we first Sanger-sequenced a 833-nt fragment of *mt-cytb*, retaining all ambiguities resulting from the putative presence of numts. We then treated all gDNA extracts with an exonuclease so as to digest linear nDNA and eliminate numts, resulting in a second dataset free of such ambiguities. We compared the first and second dataset to infer the numt sequences (the latter providing the third dataset), allowing direct comparisons of evolutionary history of the mitochondrial and numt loci. Indeed, numts being non-functional pseudogenes within the nuclear genome, they are expected to show different evolutionary dynamics in contrast with their functional counterparts in the mitochondrion [31]. To confirm that the inferred sequences correspond to numts, we looked for a reduced transversion bias compared with their corresponding mtDNA sequences, higher proportion of non-synonymous substitutions (relaxed purifying selection) and more diffuse pattern of pairwise distances [31]. Moreover, we expect numts to form a monophyletic clade in the case of only one event of transposition, as they can then diverge from their mitochondrial counterpart, as already observed in birds [32,33]. Numts may further be considered as nuclear phylogenetic markers [34,35]. We therefore investigated the relative placement of numt sequences within the mitochondrial *mt-cytb* phylogeny and reconstructed the phylogeographic history of the *P. lherminierii* complex using numt sequences. Finally, to evaluate the effects of numt contamination on the analysis of mitochondrial sequences, we compared three independent treatments to correct the ambiguities caused by numts, reflecting common practice reported in the literature (see above): (i) removing the sites with ambiguities for all individuals, (ii) removing all individuals with ambiguities and (iii) keeping ambiguities. Then, we estimated population genetic parameters on all three treatments, treating them as uncontaminated mitochondrial sequences to test whether the artefactual merging of mitochondrial and numt loci can significantly bias common metrics used in conservation genetics.

## 2. Material and methods

### 2.1. DNA extraction, PCR amplification and sequencing of mitochondrial and numt loci

**Figure 1.**
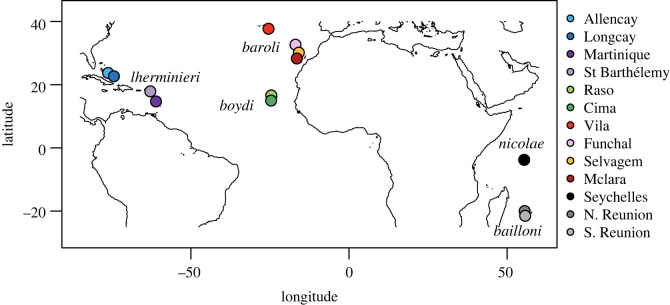
Distribution of the breeding colonies sampled for this study.

Our dataset is an extension of a companion study [30], in which blood samples from a total of 228 individuals were obtained from *Puffinus lheminieri* and *P*. *bailloni* shearwaters, totalling 13 different breeding populations. We focus here on breeding populations of the former taxon (figure 1). Extraction of total genomic DNA was carried out using NucleoSpin^®^ Tissue XS Kit (Macherey & Nagel, Düren, Germany). Samples were incubated overnight in 4 mg of Proteinase K. Purified genomic DNA was eluted twice in 50 µl of TE buffer pre-heated at 70°C. DNA concentration was measured using Nanodrop (ND 1000 model) spectrophotometry. A portion of *mt-cytb* was amplified using shearwater-specific primers designed in [30] (forward: Cytb-F1-Puf-CRI, GGCCTACTACTAGCYATACA; reverse: Cytb-R4-PUF-CRI, GTTARGATGAATAGGTTRGCG), with the proof-reading Ex-Taq polymerase (Takara Bio Europe). The PCR amplification protocol is detailed in [30].

To prevent numt co-amplification, we digested nuclear DNA with the ExonucleaseV (ExoV; NEB-M0345S), using the following protocol modified from Jayaprakash *et al*. [36]. One microgram of gDNA was heated to 70°C to inactivate any residual Proteinase K from the extraction protocol. Digestion was then carried out, adding to the sample 1X NEB4 Buffer, 1 mM ATP, 0.3 U of ExoV and 0.24 mg ml^−1^ of BSA. The mix was heated to 37°C for 48 h, followed by 30 min at 70°C to inactivate the exonuclease. We compensated the lowered PCR yield by using BSA at a final concentration of 0.24 mg ml^−1^. PCR products were sent to Eurofins (Anzinger Str. 7a/85560 Ebersberg, Germany) for purification and Sanger sequencing in both directions. Chromatograms were checked visually and sequences were aligned to our sequences using MAFFT v. 7.187 [37]. We then created two datasets of mitochondrial *mt-cytb* sequences. The first was called ‘CLEAN’, since all ambiguities due to numts were removed with the digestion protocol. The second, called ‘AMBIGUOUS’, contained ambiguities due to the co-amplification of numts in our initial PCR reactions. Before conducting further analyses, we checked that the CLEAN and the AMBIGUOUS datasets did not contain any stop codon, indel or frame-shift mutations.

## 3. Mutation patterns within numts

Numt sequences were inferred by comparing numt-contaminated (AMBIGUOUS) and uncontaminated (CLEAN) mitochondrial sequences, using a custom R script (R Core Team 2019: electronic supplementary material), resulting in the NUMT dataset. Because numts are located in the diploid nuclear genome, this technique allows the determination, within an individual, of the consensus sequence of numt alleles with mutations that are unique to the nuclear genome (i.e. the ‘divergent’ allele illustrated on electronic supplementary material, figure S1). We did not attempt phasing of the AMBIGUOUS dataset, as it is a mix of three alleles: two nuclear alleles and the mitochondrial haplotype.

The transition/transversion ratio within and among each lineage were calculated using the TiTvRatio function of the strataG R package (v. 2.0.2, [38]) and compared with Fisher's Exact Test (odds ratios are presented with their 95% confidence interval). Non-synonymous to synonymous mutation ratios (Ka/Ks) between DNA sequences from separate mitochondrial lineages were calculated [39] using DnaSP 5.10.1 [40]. Non-synonymous to synonymous nucleotide diversity ratios (*π*_a_/*π*_s_) were calculated within lineages. Both ratios were calculated for the CLEAN and NUMT datasets, using a 50 bp-wide sliding window, sliding every one bp. The same sliding window was used to calculate the Kimura 2-parameter pairwise distances [41] along the sequences using the R package ape (v. 5.2 [42]) and a custom R script. This was done for every lineage and for the pairwise comparison of each lineage, both for the CLEAN and NUMT datasets.

The number of nucleotide substitutions, at each codon position, was recorded to estimate codon position bias. Distributions obtained for the CLEAN and NUMT datasets were then compared, for each lineage, using an *Χ*^2^ test [43]. As numt sequences are non-functional, they should accumulate mutations independently from codon positions, while mitochondrial sequences should preferentially accumulate mutations on the first and third positions. Significant codon position bias among lineages is therefore indicative of multiple translocation events from the mitochondrial to the nuclear genome [31,44]. As we did not find any theoretical hypothesis about the neutral distribution of substitutions among codon positions in birds, all distributions were also compared to an equal distribution of substitutions among positions (0.3-0.3-0.3) as a null theory and compared to the observed distribution of substitutions among positions in *mt-cytb* from 16 extant Procellariiformes (0.19-0.03-0.78) [45]. Distribution inferred for each lineage was finally compared to other lineages for mitochondrial and numt sequences separately. Different codon position bias among lineages would imply that numts occurred from different functional ancestors and from independent transposition events. Codon usage bias (defined as differences in the frequency of synonymous codons) was calculated as the relative synonymous codon usage [46] using the *uco* function of the seqinr R package [47]. Frequency distributions inferred for each lineage were then compared to each other between mitochondrial and numt sequences using an ANOVA on a linear model. Distributions were compared among lineages with all mitochondrial sequences and all numt sequences. All distributions were finally compared to an equal distribution of codon usage (i.e. 1/64 for each codon). We also performed a *Z*-test of purifying selection [39] for each pair of mitochondrial/numt sequences (75 individuals). Significant different selection pressures would indicate that numt sequences are under relaxed purifying selection. The probability of rejecting the null hypothesis of strict-neutrality (dN = dS) in favour of the alternative hypothesis (dN < dS) was computed using the Nei-Gojobori method (proportion) in MEGA v. 7.0.26 [48].

Phylogenetic relationships among CLEAN and the NUMT sequences were inferred using MrBayes v. 3.2.6 [49]. The most likely model of evolution was inferred in JModeltest2 [50] using BIC and LRT, and the parameters of this model were inputted in MrBayes. Phylogenetic inference was based on 500 000 generations sampled every 1000 generations with a 25% burn-in, with four chains. Stationarity of the chains was evaluated in Tracer v. 1.7.0. The *mt-cytb* sequence for *Puffinus pacificus* (GenBank accession: AF076088.1) was used as an outgroup. The same phylogenetic analysis was conducted on the CLEAN and NUMT datasets separately, and trees were compared using the tanglegram function [51] in Dendroscope v. 3.5.10 [52]. Kimura 2-parameter (K80) pairwise distances [41] were calculated between mitochondrial and numt sequences using the R package ape [42].

## 4. Evaluating the impact of numts on statistics of diversity, divergence and differentiation

In the AMBIGUOUS dataset, two subsequent treatments were applied to correct the ambiguities in Sanger sequencing base calls, leading to two new independent datasets. First, we removed, for all individuals, the sites bearing ambiguities (hereon called the ‘SITE-LESS’ dataset). Second, we removed all the individuals presenting ambiguities (hereon called the ‘INDIVIDUAL-LESS’ dataset). In other terms, if the DNA alignment is viewed as a taxon × site data matrix, with taxa as lines and sites as columns, SITE-LESS corresponds to removing columns containing ambiguities and INDIVIDUAL-LESS corresponds to removing lines containing ambiguities.

We tested whether the distribution of ambiguous sequences among sampling populations followed a uniform distribution using the G-test [43], as implemented in the DescTools R package, and tested the correlation between the average number of ambiguous sequences within populations and the number of ambiguities within sequences using Pearson's product-moment correlation. These tests were performed in R v. 3.6.0 [53] at *α* = 0.05.

We then evaluated whether these different treatments could introduce bias in population genetics studies by computing statistics of genetic diversity, sequence divergence and population differentiation. Although numts are diploid sequences (unless mitochondrial DNA was translocated on the W chromosome), we calculated these statistics considering all data to be haploid (CLEAN, which contains haploid sequences and AMBIGUOUS, which contains a mix of haploid and diploid sequences), so as to mimic a study in which numts went undetected. For each dataset, we calculated the number of parsimony-informative sites (*S*), haplotype diversity and nucleotide diversity (*π*), using the R packages *ips* v. 0.0.11 [54] and *pegas* v. 0.11 [55]. For each population within each dataset, and for each statistic, 95% confidence intervals were computed using 1000 bootstrap replicates using the ‘sample’ function in R. NeighborNet networks were inferred using SplitsTree v. 4.14.2 [56] to further visualize the effects of ambiguities (and their treatment) on genetic diversity and differentiation within and among populations. Global *F*_ST_ was calculated for each dataset using the Weir & Cockerham [57] method implemented in the R *hierfstat* package [58]. For each dataset, 1000 bootstrap replicates were produced with each population sampled. As the *hierfstat* method does not allow us to input a model of nucleotide substitutions, we also estimated differentiation among populations by performing AMOVAs and calculating pairwise *Φ*_ST_ using Arlequin v. 3.1 [59]. While this method does not associate confidence interval with *F* statistics, it does provide statistical significance of each variance component; this was computed based on 1000 bootstrap replicates. For AMOVAs, samples were stratified into five groups, corresponding to the five nominal lineages (*lherminieri*, *boydi* and *baroli* in the Atlantic, *nicolae* and *bailloni* in the Indian Ocean), and populations (i.e. sampling localities; see map in [60]) within these groups. Pairwise Kimura 2-parameter distances, calculated for all pairs of haplotypes, were computed using the K2P model of substitution [41]. The distributions of pairwise *Φ*_ST_ obtained by the different treatments were compared to the distribution obtained with the CLEAN dataset using the Kolmogorov–Smirnov test implemented in R.

## 5. Results

### 5.1. Prevalence of ambiguities in mitochondrial mt-cytb sequences

Two sequences of 833 bp, generated with and without the exonuclease treatment, were obtained for each of the 223 individuals. Four individuals were removed from the AMBIGUOUS and NUMT datasets due to poor sequence quality (the corresponding CLEAN sequence was obtained for these four individuals, but the NUMT sequence could not be inferred with confidence). In the CLEAN dataset, 22 individuals, distributed among the five lineages, still showed ambiguities (at 37 sites) after the digestion of nuclear DNA. Because these ambiguities could be due to incomplete digestion of linear DNA, PCR amplification and/or sequencing errors, or heteroplasmy [23], we removed them from the CLEAN, NUMT and AMBIGUOUS datasets.

The final CLEAN dataset contains therefore 201 sequences, which were submitted to GenBank (Acc. Numbers OK042970-3170). In the AMBIGUOUS dataset, 75 chromatograms presented double peaks (table 1). The proportion of ambiguous sequences within a population varied from 0 (Funchal, taxon *baroli*) to 63% (*lherminieri* lineage from the Saint Barthelemy population, and *boydi* from Raso), with a median of 29%. The average number of ambiguous sites per sequence varied from 0 (in the *baroli* population from Funchal) to 61 sites (*nicolae* from the Seychelles) within populations, with a median value of 27 sites. Although the sequences with the highest number of ambiguities were from populations with the highest proportion of ambiguous sequences (e.g. *boydi* from Raso, *nicolae* from the Seychelles), these two variables were overall weakly correlated (Pearson's product-moment correlation: 0.55; *t* = 2.18, d.f. = 11, *p*-value = 0.052). The observed geographical distribution of the ambiguous sequences and the ambiguous sites were not significantly different from a uniform distribution (G-statistic = 44, d.f. = 1, *p*-value = 0.99). The CLEAN dataset presented no insertion, deletion, non-sense or stop codon following translation. The NUMT dataset presented no frame-shift mutations and no indel. However, five numt sequences showed premature stop codons following translation (Vertebrate Mitochondrial Code, NCBI genetic code table *n*. 2), suggesting drift from the original mitochondrial gene. To sum up, while the overall geographical distribution of ambiguities among bird populations was homogeneous, some numt hotspots were detected, possibly hindering biogeographic analyses.

**Table 1.** Summary of the presence of ambiguous sequences within each tested lineage and population, among the 201 sequenced shearwater individuals.

lineage	population	no. sequenced individuals	no. ambiguous sequences	proportion of ambiguous sequences	average number of ambiguous sites per sequence
*lherminieri*	Allencay	17	4	0.24	14
*lherminieri*	Longcay	17	4	0.24	22
*lherminieri*	Martinique	10	5	0.5	33
*lherminieri*	St Barthélemy	8	5	0.63	27
*boydi*	Raso	16	12	0.75	40
*boydi*	Cima	18	5	0.28	10
*baroli*	Mclara	14	7	0.50	19
*baroli*	Vila	17	5	0.29	36
*baroli*	Selvagem	4	1	0.25	10
*baroli*	Funchal	3	0	0	0
*bailloni*	North Reunion	24	7	0.29	29
*bailloni*	South Reunion	25	11	0.46	36
*nicolae*	Seychelles	28	9	0.32	61

### 5.2. Comparisons of mutation patterns within the CLEAN and NUMT datasets

The transition/transversion ratio was higher in the CLEAN dataset than in NUMT sequences within each lineage, as nine transversions were observed within the entire CLEAN dataset (versus 57 transitions; Ti/Tv = 6.33) and 30 transversions were present in the NUMT dataset (versus 103 transitions; Ti/Tv = 3.43) (odds ratio: 0.54 [0–1.13]; one-tailed Fisher's Exact Test: *p*-value = 0.09; table 2). Codon position bias was significantly different between mt and numt sequences, and it was for every lineage separately (*Χ*^2^ *p*-value < 2 × 10^−16^, table 2 and electronic supplementary material, table S2) except for *boydi* (*Χ*^2^ *p*-value: 0.99). In each lineage, except for *boydi*, the proportion of substitution in second position was non-zero in NUMT sequences, contrarily to CLEAN sequences (table 2). In the latter dataset, codon position bias was significant for *lherminieri*, *boydi* and *baroli* (electronic supplementary material, table S2ab). For NUMT sequences, codon position bias was significant for all lineages (electronic supplementary material, table S2ab). The pairwise comparisons of codon position bias between each lineage, for CLEAN and NUMT sequences, respectively, led to non-significant differences (*p*-value of the *Χ*^2^ test > 0.77), with the exception of *lherminieri*/*boydi* for the NUMT dataset (*p*-value of the *Χ*^2^ test < 2 × 10^−16^). This pair represented the highest and the lowest 2nd/3rd position ratio, respectively. None of the codon position biases differed significantly to an equal distribution. The codon position biases for the CLEAN dataset were significantly different from the observed distribution reported for Procellariiformes, contrarily to the NUMTS dataset (electronic supplementary material, table S2ab).

**Table 2.** Difference of codon bias position and transition (Ti) to transversion (Tv) ratio between mitochondrial and numt sequences.

lineage	sequence number	clean			numt			clean			numt		
		first	second	third	first	second	third	Ti	Tv	Ti/Tv ratio	Ti	Tv	Ti/Tv ratio
		position	position	position	position	position	position						
*lherminieri*	18	5	0	10	20	10	39	13	2	6,5	54	15	3,6
*boydi*	17	2	0	8	8	0	36	9	1	9	41	5	8,2
*baroli*	13	1	0	6	9	1	27	7	0	Inf	33	4	8,3
*bailloni*	18	1	0	1	11	3	41	2	0	Inf	49	6	7,1
*nicolae*	9	1	0	2	9	3	30	3	0	Inf	38	6	6,3
all lineages	75	13	1	47	31	13	77	57	6	9,5	101	25	4

All biases in codon usage were significantly different from the equal theoretical distribution (electronic supplementary material, table S2c). Within each lineage, mitochondrial and numt codon biases were not significantly different (all *p*-values > 0.05, electronic supplementary material, table S2c). More pairwise comparisons of codon biases between lineages were significantly different in the NUMT dataset (7 *p*-values > 0.05) compared to the CLEAN dataset (five *p*-values > 0.05). On the basis of the distribution of mutations across codon positions, few translocation events may have taken place, and the *boydi* population may contain recently transposed numt sequences.

The number of non-synonymous substitutions was low in the entire CLEAN dataset, leading to low Pi(a)/Pi(s) and Ka/Ks ratios along mt sequences (table 2; electronic supplementary material, figure S2a). The only exceptions being inter-lineages comparisons, with one (e.g. *boydi*-*bailloni* comparison) to five peaks (*lherminieri*-*bailloni* comparison) in the proportion of non-synonymous substitutions. More non-synonymous substitutions were present in the NUMT dataset, leading to higher Pi(a)/Pi(s) and Ka/Ks ratios (table 2; electronic supplementary material, figure S2a). Additionally, Pi(a)/Pi(s) for the NUMT dataset reveal biogeographic patterns, with, for example, a strong peak in this ratio between bases 500 and 600 for Atlantic lineages only. The sliding window of K80 pairwise distances along the sequences revealed intra-lineage substitution hotspots within the CLEAN sequences (e.g. two hotspots in *bailloni*, three in *nicolae*; electronic supplementary material, figure S2b). K80 distributions along the NUMT sequences were characterized by more divergence peaks, which were wider and higher than for the CLEAN distributions. This difference in the distribution of pairwise differences along the sequences was still visible when comparing populations having diverged for less than 300 ky, i.e. *boydi* versus *baroli* and *bailloni* versus *nicolae* [30]. The difference between the distributions of K80 distances fades when comparing all other populations (electronic supplementary material, figure S2b). The *Z*-test of selection performed on the pairs of mitochondrial/numt sequences, for each individual, showed that 35 of the 75 numt sequences (47%) had significantly different selection pressures than their mitochondrial counterparts (electronic supplementary material, table S2d) and were under less-constrained pressures. The difference between the numbers of non-synonymous and synonymous substitutions was significantly negatively correlated with the genetic distance between clean and numt sequences within an individual (Tamura-Nei 1992 distance; electronic supplementary material), confirming that the more divergent a numt is from its mitochondrial homologue, the more relaxed from purifying selection it is.

### 5.3. Evolutionary history of numts compared to mitochondrial sequences

Numts were co-amplified for 75 out of the 201 individuals used in this study (37%). The numt sequences inferred by comparing the CLEAN and AMBIGUOUS datasets were submitted to GenBank (Acc. no. OK043171-245). We analysed the modification of topology of the phylogenetic tree by comparing the branch length of both a numt sequence and the mitochondrial sequence of the same individual. As the parameters of the models of substitution inferred by the Jmodeltest analyses were different for the mitochondrial and the numt datasets, comparisons of branch lengths between these datasets (HKY with *κ* = 26 and 13 for the mt and numts, respectively) should be done with caution. Twenty-nine of the numt sequences (39%) were placed close (less than 10% difference compared to the length of the branch calculated from the CLEAN alignment) to their associated mitochondrial sequence in the phylogenetic tree (electronic supplementary material, table S3). The average pairwise distance between each of these numt sequences and their associated mitochondrial sequence was 0.76%. Ten numt sequences (13%) were in the same clade as their mt corresponding sequence but closer to the root of the tree with an average genetic distance of 0.33%. Conversely, 24 numt sequences (32%) showed branches longer than their mitochondrial counterparts, with an average genetic distance of 0.88%. These values were superior to the intra-lineage average pairwise distance (0.13% to 0.34%) but inferior to the inter-lineage distance (0.95% to 3.83%) found with the mitochondrial sequences (electronic supplementary material, table S4).

**Figure 2.**
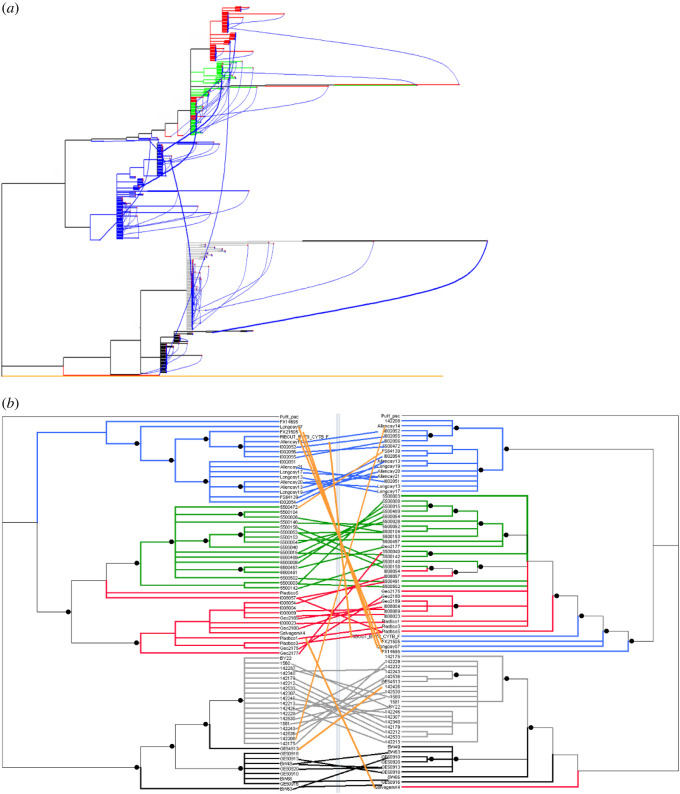
Phylogenetic relationships among mitochondrial and numt sequences. (*a*) Pooled mitochondrial and numt sequences. Tree tips are coloured according to lineages (*lherminieri* in blue, *boydi* in green, *baroli* in red, *nicolae* in black, *bailloni* in grey and *P. pacificus* outgroup in orange). At each tip, the sequence origin is provided as a coloured dot (mitochondrial as a blue dot, numts as red dots). Mitochondrial and numt sequences retrieved from a single individual are linked by a curved blue line, the width of which is proportional to the genetic distance between the aforementioned sequences. Posterior probabilities greater than 0.95 are indicated. (*b*) Separately analysed mitochondrial (left) and numt (right) sequences. Tree tips are coloured according to lineages as above. Segments link sequences from a single individual. Orange lines show discordant mitochondrial/numt phylogenetic positions while concordant sequence pairs are coloured according to their lineage. Only the individuals presenting different mitochondrial and numt sequences (genetic distance ≠ 0) were used. Posterior probabilities greater than 0.95 are shown for (*b*).

The phylogenetic position of numts and uncontaminated mitochondrial sequences were discordant in 14 individuals (19%; average genetic distance between the numt and mitochondrial sequence of 2%), numt sequences falling outside of their expected mitochondrial clade (figure 2*a*,*b*). Only two of these discordances corresponded to a shift in oceanic basin, a *bailloni* individual presenting a numt sequence placed in *lherminieri* and a *baroli* individual presenting a numt sequence in the Indo-Pacific clade. Within the Atlantic lineages, five *lherminieri* individuals presented numt sequences in one of the two east Atlantic lineages: four *baroli* numt sequences were found in the *boydi* clade, while one *boydi* numt sequence was found in the *lherminieri* clade. Within the Indo-Pacific clade, two *nicolae* sequences were found in the *bailloni* clade. The tree inferred exclusively from numt sequences showed, however, the same topology as the *mt-cytb* tree, except for the individuals listed above (figure 2*b*).

### 5.4. Impact of the different strategies to deal with numts

**Figure 3.**
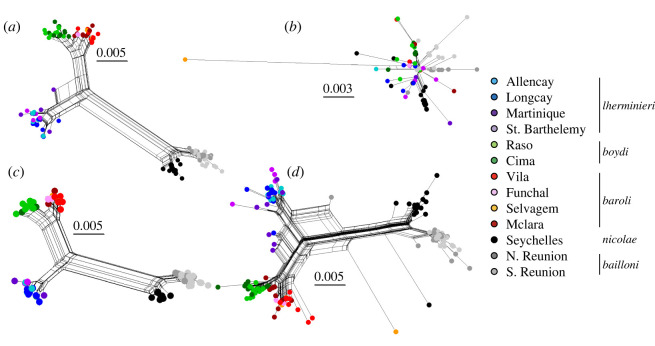
Haplotype networks for the five datasets and all individuals. (*a*) CLEAN dataset. (*b*) SITES-LESS dataset. (*c*) INDIVIDUAL-LESS dataset. (*d*) AMBIGUOUS dataset. The scale bars show how the length of a branch translates in sequence divergence. The unit is divergent nucleotides divided by the length of the sequence analysed.

A total of 113 sites (representing 14% of the sequence) were lost by removing all the sites presenting double peaks. However, the number of parsimony-informative sites dropped drastically between the CLEAN dataset and the SITE-LESS dataset, with 81% of the informative sites being lost overall (table 3), 50–100% depending on the population (electronic supplementary material, table S6). This indicates that the divergence of numts from original mitochondrial copies (i.e. intra-individual divergence between homologue and paralogue) and the divergence among mitochondrial copies (i.e. inter-individual divergence among homologues) involve the same point mutations, which is consistent with the sliding window analysis of average pairwise distance (electronic supplementary material, figure S2b). Indeed, of the 113 polymorphic sites between the numt and mitochondrial sequences, 60 (52%) were common to the mitochondrial and numt sequences, 43 (37%) specific to numts (with 26 singletons) and 12 (10%) specific to mitochondrial sequences (with 10 singletons). This loss of information impacted every statistic comparing the SITE-LESS dataset to the CLEAN dataset. The drop of haplotype and nucleotide diversity was significant both among (table 3) and within populations (electronic supplementary material, table S6). The same result is visible on haplotype networks of the SITE-LESS dataset, where almost all genetic structure was lost, compared to the CLEAN network (figure 3). Most pairwise *Φ*_ST_ values were non-significant in the SITE-LESS dataset and consistently lower than in the CLEAN dataset (table 4; electronic supplementary material, table S6, Student's test: *p* < 2.2 × 10^−16^, *t* = 11.8). Moreover, most of the genetic variance was due to within-lineage differentiation, while inter-lineage differentiation was 70% lower than found for the CLEAN dataset (table 4). No divergence times could be estimated since no significant structuration among lineages emerged.

**Table 3.** Diversity statistics. *S* represents the number of parsimony-informative sites, *h* the haplotype diversity and π the nucleotide diversity. 95% confidence intervals (within brackets) were calculated based on 1000 sample bootstraps. Dataset dimensions are provided in terms of sequences × nucleotide positions.

dataset	*S*	*h*	*π*	dimensions
CLEAN	59 [57–66]	0.95 [0.93–0.96]	0.0236 [0.0229–0.02383]	201 × 833
SITES-LESS	11 [9–24]	0.82 [0.37–0.85]	0.0018 [0.00157–0.00215]	201 × 720
INDIVIDUAL-LESS	54 [54–63]	0.95 [0.93–0.96]	0.0238 [0.02307–0.02411]	140 × 833
AMBIGUOUS	59 [59–76]	0.94 [0.93–0.96]	0.0226 [0.02177–0.02312]	201 × 833
NUMTS	59 [58–74]	0.94 [0.93–0.96]	0.0226 [0.02186–0.02309]	201 × 833

**Table 4.** Global differentiation statistics. Global *F*_ST_ was calculated both by the Weir and Cockerham method implemented in the R *hierfstat* package with 95% CI calculated from 1000 samples bootstrap and by an AMOVA in Arlequin. *V*a, *V*b and *V*c represent the percentage of variation among groups, among populations within groups and within populations of the AMOVA, respectively.

dataset	global *F*_ST_ hierfstat	global *Φ*_ST_ Arlequin	*V*a	*V*b	*V*c
CLEAN	0.87 [0.88–0.92]	0.92	90.49***	1.75***	7.76***
SITES-LESS	0.69 [0.64–0.79]	0.37	27.98***	8.27***	63.75***
INDIVIDUAL-LESS	0.89 [0.89–0.9]	0.92	91.02***	1.59***	7.39***
AMBIGUOUS	0.77 [0.75–0.83]	0.92	90.19***	1.47***	8.34***
NUMTS	0.89 [0.88–0.92]	0.91	89.42***	1.72***	8.85***

Conversely, the other strategies (AMBIGUOUS and INDIVIDUAL-LESS) showed no significant change for any genetic diversity indexes (i.e. parsimony-informative sites, haplotype and nucleotide diversity; table 3). However, in the INDIVIDUAL-LESS dataset, a significant loss of diversity was observed for the populations showing the highest proportion of ambiguous sequences: Martinique and St Barthélemy from the *lherminhieri* lineage and Raso from the *boydi* lineage (table 1; electronic supplementary material, table S6). Despite this loss of diversity, divergence at the oceanic and lineage scale is retained, as shown by the network analyses where the closest results to CLEAN datasets were obtained with the INDIVIDUAL-LESS dataset (figure 3). The proportion of variance explained by inter-lineage variation was higher than 0.05% compared to CLEAN (table 4). However, lower diversity was associated with lower levels of population differentiation in the pairwise *Φ*_ST_ analysis, where most values involving Martinique, St Barthélemy or Raso populations decreased or were non-significant in the INDIVIDUAL-LESS dataset (electronic supplementary material, table S7, Student's test: *p* = 1).

When comparing the AMBIGUOUS and the CLEAN datasets, all diversity (table 3) and differentiation (table 4) statistics were not significantly different from each other, both among and within groups. The genetic variances explained among groups (*V*a) and among populations within groups (*V*b) were >1%lower for AMBIGUOUS compared to CLEAN. The only exception was the global *F*_ST_ analysis obtained by *hierfstat*, from which the AMBIGUOUS value was significantly lower than the CLEAN values (table 4). This result was neither found in *Φ*_ST_ pairwise analyses nor in the global *Φ*_ST_ analysis in the Arlequin software (table 4 and electronic supplementary material, table S7, Student's test: *p* = 0.34). This could be due to a difference of treating the missing data between the two methods.

## 6. Discussion

### 6.1. Evolution of numt sequences

The sequences inferred by comparison of the CLEAN and AMBIGUOUS datasets present several properties expected from numts. First, the fact that most SNPs disappear when linear DNA is enzymatically digested indicates that these sequences are of nuclear origin. They show significantly lower transition/transversion ratio and codon position bias than their mitochondrial counterparts and significantly more non-synonymous substitutions. The transition/transversion ratio (values from *mt-cytb* in birds ranging from 1 to infinity [61]) has been shown to be lower in numts sequences than in their mitochondrial counterparts (e.g. in birds [62]). Finally, about a half of the NUMT sequences showed a significant departure from the purifying selection acting on their mitochondrial counterpart; the more divergent paralog/homologue *mt-cytb* sequences were, the stronger this departure was. All of this is consistent with the fact that these sequences are under relaxed selection compared to their mitochondrial counterpart [31].

Weaker selection pressure makes more likely the appearance of non-synonymous substitutions, e.g. transversions in codon first or second positions, which leads to higher average pairwise distances within populations. However, following [33], the mutation rate of mitochondrial DNA is expected to be higher than nuclear DNA, including numts, in birds. The fact that we found more mutations among numt sequences than among mitochondrial sequences could be explained by the co-amplification and sequencing of multiple numt copies within individuals. Additional work to estimate the number of nuclear *mt-cytb* loci would help resolve this issue.

These numts bear phylogenetic information, similar but not identical to the mitochondrial *mt-cytb* sequences. Three (*lherminieri*, *nicolae* and *bailloni*) of the five mitochondrial lineages were recovered as monophyletic in the numt tree. However, the east Atlantic *baroli* and *boydi* mitochondrial lineages appeared polyphyletic based on numts, as for other nuclear markers tested thus far [30]. Moreover, multiple individuals displayed mito-nuclear discordance (detailed below). These similarities and discordance between the mitochondrial and the numts phylogenies can be explained by multiple translocation events that occurred from the mitochondrial to the nuclear genome at different times during the evolution of the species complex. This is consistent with the observation that numts do not form a monophyletic clade [31], and the low divergence separating most numts from their mitochondrial counterpart indeed suggests recent translocations followed by slow accumulation of mutations on nuclear *mt-cytb* copies [32]. Only two individuals bear numt signatures discordant with the mitochondrial clade at the ocean basin level (i.e. one Atlantic individual with an Indo-Pacific numt, and one Indo-Pacific individual with an Atlantic numt). These copies may have resulted from mitochondrial transposition events that occurred before the diversification of Atlantic and Indo-Pacific *Puffinus* lineages, at least 1 My ago [30]. This is theoretically possible, since 10–14 My translocation events have been reported [63–65], and suggests that not all events of transposition are recent. This could also explain the fact that a signature of divergence between *boydi* and *baroli* is not found in the numts sequences if the transposition event occurred before their mitochondrial divergence.

The numts sequences show similar phylogenetic patterns to other nuclear loci. Indeed in a previous study [30] performed on the same sample set, we have shown that single-copy nuclear markers (*βfib*, *csde*, *irf2*, *pax*, *rag1* and *tpm*) do not allow the discrimination of the *boydi* and *baroli* lineages, as found here with the numt sequences. We have also shown that several individuals presented mito-nuclear discordance [66] for at least one out of the six nuclear markers tested. For example, two individuals of *boydi* (NE Atlantic) showed a *βfib* (nuclear) sequence placed in the Indo-Pacific clade (see electronic supplementary material, table S5a). These two particular individuals presented numt sequences that were basal to the East Atlantic population (this study). Six other individuals showed a nuclear sequence placed in a discordant clade relatively to their geographic origin in [30] and a numt sequence discordant from the mitochondrial sequence (electronic supplementary material, table S5a). Similarly, based on *βfib*, *pax*, *irf2* and *tpm*, an individual had been identified as a possible hybrid between the *nicolae* and *baroli* lineages. In the present study, a numt sequence was found in the Indo-Pacific clade, whereas its mitochondrial counterpart and its geographic origin indicate a *baroli* origin. The proportion of individuals being discordant both for numt sequences and for at least one nuclear marker (i.e. 25% of discordant individuals for *pax* are also discordant for numts) is similar to the proportions of individuals discordant for two non-numt nuclear markers (e.g. 25% of discordant individuals for *pax* are also discordant for *βfib*, see electronic supplementary material, table S5b). Moreover, the patterns of discordance for numt sequences are similar to what we observed for nuclear markers [30] and could be explained by the same processes. We recently proposed that the aforementioned nuclear markers may reveal recent introgression and/or incomplete lineage sorting (ILS) in this species complex. The same processes could affect a single nuclear copy of *mt-cytb*, resulting in mito-numt discordance. Numts, as part of the nuclear genome, are likely submitted to some of the same processes as nuclear loci used for phylogenetics and phylogeography. We thus conclude that the polyphyletic placement of 37% of the numts sequences could be the result of recent introgression and/or ILS. These processes could be sorted out using genome-wide genetic markers [67]. Finally, numts may be helpful in phylogeographic study, e.g. [68]. Numts have previously been used to detect hybridization in mammals [69] and have proved useful as phylogenetic markers [34,70]. Focusing on Darwin's Finches, Sato *et al.* proposed that mtDNA be used to resolve terminal nodes and numt deep nodes, as the former is more prone to saturation than the latter [62]. Numts can bring supplemental temporal information if their translocation to the nucleus occurred before evolutionary splits of interest (e.g. [28,33]). However, numt characteristics, such as abundance, vary across taxa, even across populations [31]. Here, the phylogenetic relationships among mitochondrial lineages inferred from *mt-cytb* numts corroborates the result from six nuclear markers suggestive of introgressive hybridization among these lineages [30]. In mammals and birds, the nuclear genomes, including numts, evolve much slower than mitochondrial DNA [33]; short numt sequences will show little inter-population divergence and can therefore offer a snapshot in time of ancient mitochondrial DNA genetic structure. Inadvertent numt amplification can lead to incorrect phylogenies, for example, by inferring a supplemental monophyletic group, which in fact corresponds to paralogues [33]. Particular attention should therefore be paid to the amplification of numts with mitochondrial markers, which could bias phylogenetic inferences but also population genetics analyses.

### 6.2. Impact of numts co-amplification on mitochondrial genetics analyses

Diversity and differentiation analyses on *mt-cytb* were inferred in a companion study, on the same individuals, using additional mitochondrial (*mt-cox1* and CR) and nuclear (single-copy introns: *βfib*, *csde*, *irf2*, *pax*, *rag1* and *tpm*) markers [30]. The evolutionary patterns inferred using *mt-cytb* are consistent with the analyses performed on *mt-cox1* and CR (each of the five nominal groups form a monophyletic clade, no significant genetic structure within these groups). However, we have shown that nuclear markers were less resolutive within this complex (e.g. separation of Indian and Atlantic Ocean lineages, East and West Atlantic lineages, no genetic structure evidenced within these groups). We proposed that the discordance between mitochondrial and nuclear data was due to ILS and/or introgression events occurring within this complex. These processes may have an impact on the numt sequences as well.

Numt and mitochondrial datasets had similar levels of genetic diversity, but some numt sequences were more divergent from each other (45% of raw *p* distance > 0.5%) than what was observed for mitochondrial sequences (e.g. average *lherminieri* intra-lineage raw *p* distance = 0.4%, electronic supplementary material, tables S3 and S4). Such sequences will bring high bias in analyses, artificially raising diversity within populations and accentuating differentiation (when using genetic distance-corrected statistics like *Φ*_ST_) among populations. Only five of the numt sequences (7%) contain stop codons; while verifying the absence of stop codons in mitochondrial protein-coding sequences is essential, it does not guarantee weeding out all existing numts.

Mutations along numt sequences can coincide with the position of informative mutations on the mitochondrial locus, hence blurring the phylogeographic signal. The same pattern was found in ambiguities due to the duplication of the CR (60% of informative sites contain ambiguities in the CR, [30] and data not shown). Hence, removing the sites comprising ambiguous data leads to a massive loss of information and must be avoided. In their study, Kerr & Dove [25] trimmed the alignment in the 3′ extremity of the CR sequences, which seemed have little effect on their final results, since the analyses inferred with these sequences were similar to the analyses of the complete *mt-cox1* sequences.

In our dataset, the SITE-LESS treatment (i.e. removing ambiguities) had the most severe impact on informative sites, especially in populations with greater than 30% sequences bearing ambiguities, while INDIVIDUAL-LESS strategy had little effect. Genovart *et al*. [26] similarly used the latter method on well-sampled populations, and with a proportion of removed individuals less than 30%, hence their results are likely little biased, but results from studies with lower sampling (less than 10 individuals per population) should be treated with caution. Using ambiguities led to reduced genetic information, although not significant in our dataset. Evidently, separating mitochondrial sequences from their paralogs is the best course of action. In this study, enzymatic digestion of linear DNA has proven to be an efficient and inexpensive strategy for doing so. If this is impractical, however, we found that using either the INDIVIDUAL-LESS or AMBIGUOUS treatments of ambiguous datasets will minimize the bias in analyses of genetic diversity, population divergence and differentiation.

### 6.3. Avoiding the coamplification of numts

Aside from the intrinsic value of studying numts and their evolution, it can be desirable to avoid numts altogether. This can be done by carefully choosing tissue rich in mitochondrial DNA, as proposed to avoid numt coamplification in humans [71]. Biological samples other than blood are therefore best for mtDNA analysis in birds [18]. Unfortunately, blood is by far the most used tissue for DNA analysis (60% of studies, electronic supplementary material, table S1); it is therefore important that, if researchers can only rely on this tissue type for DNA analysis, other safeguards be implemented to avoid numts.

Digestion of linear DNA is an efficient way of removing numts when only orthologous mtDNA is desired [30] but has the drawback of requiring a substantial amount of DNA (starting with 1 µg of gDNA usually resulted in trace amounts of circular mtDNA post-exonuclease digestion). If mtDNA is fragmented, it will be digested as nuclear DNA. This technique is therefore not applicable to older samples such as museum specimens.

Designing and using specific primers have been proposed to prevent coamplifying numts [28]. This strategy was empirically tested by Moulton *et al.* [72] for orthopteran insects, for which numt co-amplification is rampant. They found that primer specificity does not correlate with lower numt coamplification, numt age or background noise in Sanger sequencing of PCR products (ambiguities). Our study originally started using primers designed specifically for the *Puffinus* genus, based on publicly available sequences [30]; this strategy did not prevent us from hitting numts. Likewise, Deane [73] used copy-specific primers to amplify the CR and found ambiguities nevertheless. Finally, some studies in our review used internal primer yielding very small amplicons (less than 200 bp; electronic supplementary material, table S1). This strategy can be used to amplify DNA from degraded samples at the risk of amalgamating mitochondrial and nuclear gene copies.

A third, very efficient safeguard is to disentangle mitochondrial and nuclear copies after the PCR step. While cloning of PCR products is an efficient way of cataloguing orthologous and numt haplotypes (e.g. [74]), it remains costly in terms of time and money. Another technique to catalogue numts is second-generation sequencing of PCR production without cloning. Illumina sequencing of PCR products as proposed by Shokralla *et al*. [75] might therefore be an economical way of testing for the presence of numts when DNA is scarce (e.g. when working with museum specimens).

Finally, data analysis is our last line of defense against numts. Searching for stop codons and indels in sequences will get rid of some undesirable data but is not sufficient for eliminating all co-amplified numts [72]. Some authors also looked at deviation from expected transition to transversion ratios as a way to detect numts (e.g. [76]). Indeed, in our case, the overall Ti/Tv ratio of numts was significantly different from mt copies. However, none of these analytic safeguards will be efficient against recently translocated pseudogenes.

## 7. Conclusion

Despite the recent development of so-called ‘next-generation’ molecular markers for molecular systematics, phylogeography and phylogenetics (e.g. [77]), mtDNA remains a resourceful tool in evolutionary biology [10]. In phylogenetics, for instance, mitochondrial DNA still holds the best signal/cost ratio [78]. As any marker, mtDNA has advantages and drawbacks [10], one being the coamplification of nuclear pseudogenes that can lead to significant noise in evolutionary studies, as exemplified here for shearwaters. Preventing such problems can be avoided by carefully designing DNA sampling (e.g. avoiding mt-poor tissues and removing nuclear DNA), amplification (using mt-specific primers in combination with stringent cycling conditions), sequencing (Sanger sequencing of PCR clones or use of second-generation sequencing) and data analysis (transition/transversion ratios, detection of spurious point mutations, premature stop codons, indels, as well as genetic distance between haplotype and phylogenetic placement).

## Data Availability

DNA sequences are available on Genbank (Accession no.: OK042970-3170 for the CLEAN dataset, OK043171-245 for the NUMT dataset). The data are provided in the electronic supplementary material [79].
